# Supplementary material for: Is retina affected in Huntington’s disease? Is optical coherence tomography a good biomarker?
Source: PLoS One. 2023 Feb 24;18(2):e0282175. doi: 10.1371/journal.pone.0282175 (PMC9955964; doi:10.1371/journal.pone.0282175)
Supplement: S1 Table — HD–Huntington’s disease patients, HC–healthy controls (DOCX) [file pone.0282175.s001.docx]

Supplementary Table 1: Comparison of published studies and their findings in the main OCT parameters.

| **Study** | **N** | **RNFL temporal** | **RNFL superior** | **RNFL peripapillary** | **RNFL**  **Inferior** | **RNFL**  **nasal** | **RNFL total** | **Macular volume** | **Choroid** |
| --- | --- | --- | --- | --- | --- | --- | --- | --- | --- |
| Kersten et al [7] | 26 HD 29 HC | Reduced | Normal | Normal | Normal | Normal | Normal | Normal | - |
| Haider et al [8] | Total 51 | Normal | | | | | | Reduced |  |
| Gatto et al [9] | 14 HD 13 HC | Reduced | Reduced | - | Normal | Normal | Normal  (Stage III reduced) | - | - |
| Andrade et al [10] | 8 HD 8 HC | Normal | Normal | Normal | Normal | Normal | Normal | Normal  (TMS correlation) | Reduced macular choroidal thickness |
| Di Maio et al [11] | 32 HD 26 HC | - | Normal | - | Normal | - | Normal |  | Reduced central choroidal thickness |
| Dusek | **41 HD 41 HC** | Normal | - | - | - | - | Reduced (small effect size) | Reduced (small effect size) | - |

HD – Huntington’s disease patients, HC – healthy controls.
