# Supplementary material for: Is retina affected in Huntington’s disease? Is optical coherence tomography a good biomarker?
Source: PLoS One. 2023 Feb 24;18(2):e0282175. doi: 10.1371/journal.pone.0282175 (PMC9955964; doi:10.1371/journal.pone.0282175)
Supplement: Attachment — Submitted filename: Response to Reviewers.docx [file pone.0282175.s002.docx]

Dear reviewers,

we are pleased to read your comments and insights to our work. You have provided us with key feedback that help us do better research. We had to deal with some issues by stating them in the limitations section. However, we still think that our findings are worth as a publication, since it contradicts older studies and has the largest sample size. We have addressed the comments by making the conclusion more appropriate to our findings.

Our response to comments is as follows (comments stated in italic type, our response in boldface):

*The authors evaluate retinal OCT findings as a possible biomarker for HD severity and progression. This is the largest number of patients included in a retinal evaluation of HD, and thus deserves a comprehensive analysis of the different layers, in addition to the RNFL and RGC complex. The temporal RNFL changes they report are small. And there is enough variability that 4 referenced studies were evenly divided in terms of these changes. The expansion repeats (~44) are in agreement with adult onset/complete penetrance, but do not take into consideration more severe/juvenile forms, nor does it consider late disease. However, this is a minor consideration, particularly since the authors are talking about identifying biomarkers for HD.*

**We added the fact that this study included only adult Huntington's disease patients to the study limitations.**

*To compare with expansion repeat numbers, age and disease duration, the authors use SD-OCT, contrast sensitivity (for acuity) and D15 color testing. Scotopic/photopic testing was not performed. This is of interest because the authors quote a previous functional study released only as an abstract (Johnson et al, 2012) which suggested inner retinal dysfunction that may be unassociated with RGCs.*

**We added to the study limitations that the scotopic/photopic testing was not performed.**

*Previous HD models have shown retinal degeneration, but it is unclear whether the changes in the early models (in the fly and mouse) are true indicators of the disease, since their expansion repeats were huge, and may have contributed to unusual retinal degeneration patterns similar to retinitis pigmentosa.

Previous reports have shown a decline in contrast sensitivity in HD patients, suggesting a loss of outer retinal, or other outer-retina-associated function, but the majority of studies have really focused on inner retinal function; in particular retinal ganglion cells (RGCs) and their axons The authors found a loss of RNFL thickness in the termporal region, but this was a trend, and not significant. They conclude that there are global RNFL changes, but not suitable as a biomarker, and that retinal parameters are not appropriate for monitoring HD disease progression. This is a considerable leap, since what they are really saying is that a) retinal SD-OCT at its present development is not suitable. B) the retinal parameters they looked at are not suitable. C) D-15 color testing is not a good damage marker of retinal parameter changes in HD. All are valuable considerations.*

**We have changed the conclusion section accordingly.**

*I question then why they found changes in total macular volume in HD patients, which does seem to be significant, and which does suggest some associated retinal function besides RNFL. It also suggests that there may be changes in layers other than the RNFL.*

**We performed a post-hoc analysis showing that the difference in macular volume was a false positive and was appropriately corrected by the false discovery rate correction. We showed that there is no interaction between age and triplet expansion length in the effect on macular volume. The rate of macular volume loss with aging is even (not significantly) steeper in the control group than in the patients group. These analyses were added to the discussion section.**

*In short, the authors need to utilize their data to perform a more detailed analysis of the different layers of the retina. This would make it more comprehensive, and more accurate for the conclusions that they draw (that OCT is useless in identifying HD retinal biomarkers).*

**Analysis of the individual retinal layers of the macula requires a high-quality scan. Examination of the retina using OCT had to be adapted to involuntary movements and the ability to cooperate during the examination in patients with HD. In many patients, the quality of scans does not allow reliable segmentation of the retinal layers in the macula – because of their involuntary movements.**

**In any case, we think that the examination that we cannot reliably perform in these patients is not a suitable method for studying the progression of the disease.**

**We have changed the limitations section to state these issues and the conclusion section accordingly to make statements about the performed methods only.**

**All the changes in the text were highlighted in yellow.**

Additionally, during the review process, funding of our work changed, so we edited the funding sources slightly.

Thank you for reviewing our work. We look forward to your opinion on our changes.

Yours faithfully,

Pavel Dusek
