# Supplementary material for: Is retina affected in Huntington’s disease? Is optical coherence tomography a good biomarker?
Source: PLoS One. 2023 Feb 24;18(2):e0282175. doi: 10.1371/journal.pone.0282175 (PMC9955964; doi:10.1371/journal.pone.0282175)
Supplement: Attachment — Submitted filename: REview Jan 2023.docx [file pone.0282175.s003.docx]

The authors have replied to my review with some limited changes. My major concern is that it is important to recognize that while the SD-OCT test has wide capabilities, it is limited by the problems of the current ability to examine. I have also evaluated HD patients, and their shake/eye movements make it difficult to get a ‘lock on’ and high quality scans. Thus, it is necessary to distinguish the problems of CURRENT technology, and not simply say that OCT cannot be used (it cannot be used without more refinements). Otherwise, people may simply quote their paper to show ‘it cannot be done’, and suppress future research. Thus the suggested changes:

1. abstract: in conclusions: ‘Current’, not ‘These
2. Discussion/third paragraph: add ‘**Changes** in male maculae are….
3. Discussion/last paragraph/last sentence: ‘In many patients**, due to the limitations of the current technology**, the quality of the scans….
4. Conclusions:
5. However, it seems that, **within the limits of current technology and software**,
