# Supplementary material for: Is retina affected in Huntington’s disease? Is optical coherence tomography a good biomarker?
Source: PLoS One. 2023 Feb 24;18(2):e0282175. doi: 10.1371/journal.pone.0282175 (PMC9955964; doi:10.1371/journal.pone.0282175)
Supplement: Attachment — Submitted filename: Response to Reviewers_2023-02-08.docx [file pone.0282175.s004.docx]

Dear prof. Bernstein,

we thank you for your time and for your suggested changes to improve our research. We incorporated them fully into the manuscript:

1. In the Abstract, we changed ‘*These retinal parameters are not appropriate for monitoring HD disease progression.*’ to ‘*Current retinal parameters are not appropriate for monitoring HD disease progression.*’
2. In the Discussion, we changed ‘*Male maculae are on average greater than female maculae.*’ to ‘*Changes in male maculae are on average greater than in female maculae.*’
3. In the Study Limitations, we changed ‘*In many patients, the quality of the scans does not allow reliable segmentation of the retinal layer in the macula.*’ to ‘*In many patients, due to the limitations of the current technology, the quality of the scans does not allow reliable segmentation of the retinal layer in the macula.*’
4. In the Conclusion, we changed ‘*However, it seems that the magnitude of these abnormalities is very small and not clinically useful.*’ to ‘*However, it seems that, within the limits of the current technology and software, the magnitude of these abnormalities is very small and not clinically useful.*’

Additionally, we included the Figure 1 description into the manuscript file, as requested by the Editorial Office.

We think that the revised manuscript is now more balanced in terms of study limitations and possible suggestions for future research. We look forward to your opinion on the manuscript.

Yours sincerely,

Pavel Dusek
